# What is it like to be a sport school student-athlete? A mixed method evaluation of holistic impacts and experiences

**DOI:** 10.1371/journal.pone.0289265

**Published:** 2023-11-30

**Authors:** Ffion Thompson, Fieke Rongen, Ian Cowburn, Kevin Till

**Affiliations:** 1 Carnegie School of Sport, Leeds Beckett University, Leeds, United Kingdom; 2 Leeds Rhinos Rugby League Club, Leeds, United Kingdom; Mugla Sitki Kocman University: Mugla Sitki Kocman Universitesi, TURKEY

## Abstract

Assessing the holistic impact of student-athletes within sport schools is important due to the increasing popularity of sport school programmes, the likelihood that most youth athletes do not ultimately succeed in their sport and the multiple and wide ranging positive and negative impacts associated with intensified youth sport. Therefore, this study, using a mixed method design, aimed to evaluate the ‘in-time’ holistic impacts and experiences of being a sport school student-athlete. Five data collection methods (i.e., online questionnaire, physical fitness testing battery, academic assessments grades, injury data and log diaries) were used to assess athletic, academic, psychological and psychosocial holistic impacts and experiences of 83 student-athletes from one sport school in the United Kingdom (UK). Due to the mixed method approach, a triangulation design was used whereby quantitative and qualitative data were firstly analysed separately and then integrated and presented together. Overall, the findings demonstrated there were a multitude of positive impacts and experiences associated with being a sport school student-athlete. These included: high average academic attainment, satisfaction with academic support, sport competence, all-round sport development, higher general and sport specific recovery than stress, inter- and intra-personal development, social support, positive peer and parent relationships and dual career motivation. However, impacts and experiences of concern were also apparent including: participation in sport external to the school context, difficulty balancing education and sport, academic lessons missed, injury, fatigue, lack of free time, extra-curricular and social sacrifice, social intensity and body image concerns. Large inter-individual variability was demonstrated across all data analyses highlighting the variable nature of the impacts and experiences of being a sports school student-athlete. Overall, sport schools have the potential to promote many positive holistic impacts, however stakeholders need to be aware, monitor and mitigate the potential negative impacts. Flexible development programmes, individualised support and student-athlete monitoring are essential features required of sport schools to ensure healthy and holistic development for all sport school student-athletes.

## 1. Introduction

Over the last two decades, youth sports training programmes have intensified. This intensification has resulted in a significant increase in the global distribution of sporting talent identification and development systems (TIDS) across and within sports [1]. Although TIDS are believed to increase the likelihood of athletic success [2, 3], they can be competitive, pressurised, stressful and ego-based environments [4, 5], which could threaten athletes’ health (e.g., injury, psychological overload; [6]). As such, the moral justification and healthiness of entering youths into intensive training environments has increasingly been questioned [7, 8]. As a result, there is a growing call for TIDS to take a holistic approach that promotes healthy performance and positive personal development and experiences [e.g., 9, 10].

Youth athletes involved in TIDS also have to continue their formal education. Worldwide, the term ’dual career’ (DC) has been introduced to aid youth athletes with the specific challenge of balancing sport and education [11]. Although recent research has demonstrated the benefits of combining sport and education [12, 13], it can be a challenging proposition for most young athletes [14]. Combining sport and education requires extensive time commitment [15], which can result in fatigue, stress, a loss of motivation, a lack of opportunities to participate in activities outside of sport or education, as well as athlete overload and increased injury risk [e.g., 16, 17]. These potential negative effects lend weight to the increased need for a holistic and long-term approach toward DC development [18, 19].

In light of the need for a holistic approach towards DC, the Holistic Athletic Career model [20] has been recognised as one of the most comprehensive frameworks [e.g., 21], describing an athlete’s career across multiple developmental dimensions (i.e., academic/vocational, athletic/physical, psychosocial and psychological). This model has been extensively used in research studies within sport to guide data collection about the athlete as a whole person [e.g., 22]. By advocating a holistic approach, DC programmes are not only nurturing successful athletes but also developing competencies and skills that allow them to cope with the challenges they face in sport and other life domains (e.g., education, social; [23–25]). Schools have been proposed to be important DC environments, where youth athletes have access to coaches and teachers that can foster holistic development [26]. A particular example of a DC development environment that may cater for youth athletes’ holistic development is a sports-friendly school, defined as a *“regional educational institution*, *who permit elite sport or align themselves with elite sport to provide academic flexibility for athletes to train and compete in their own sporting environment”* [27 p. 140].

Sport-friendly schools are integrated school environments for sport and education (and sometimes boarding), where educational classrooms, sports facilities and accommodation buildings are all in one location [28]. To accommodate holistic development, sport-friendly schools have numerous athletic (e.g., high-quality facilities and high volume/frequency of training), academic (e.g., extra tutoring, dedicated study hours, high academic workload) and psychosocial/psychological (e.g., pastoral services and support network) features [29]. Thompson et al., [29] review highlights a multitude of immediate, short- and long-term, positive and negative impacts and outcomes associated with being a sports school student-athlete that stakeholders (e.g., teachers, coaches and parents) should be aware of when designing, implementing and evaluating sports school programmes [29]. However, whilst research studies have examined individual components of holistic athletic development, limited research simultaneously evaluates all four holistic domains (i.e., academic, physical/athletic, psychosocial and psychological; [20]). Therefore, more multidimensional studies assessing the holistic development of student-athletes at sports schools are warranted. Furthermore, the majority of sports school studies are conducted within Northern Europe (e.g., Denmark, Norway, Sweden, Finland; [29]), which tend to have state sponsorship and specific policy approaches toward the dual-careers of student-athletes [30]. Conversely, in the United Kingdom (UK), developing a sports-friendly school is primarily a matter of individual schools. It is often pursued as part of a strategy to create a distinct identity [31]. As a result, it is important to investigate further the individual contexts of sports-friendly schools within the UK specifically.

Recently, Thompson et al. [28] qualitatively explored the features of a sport-friendly school in the UK and their impact on the holistic development of student-athletes from the perspective of the student-athletes, coaches and teachers. The findings highlighted a wide range of potential impacts, but overall, the student-athletes tended to receive a well-rounded, balanced experience of sport, education and psychological/psychosocial development. However, balancing the high workload of sport and education, alongside other factors (e.g., playing sport externally), did present challenges. Therefore, it is important that sport-friendly school programmes promote positive and ameliorate negative impacts through the design of an appropriate learning environment that emphasises the holistic development of youth athletes to ensure a system "worth" [32]. In order to ensure such “worth” it is necessary to carefully monitor a range of factors, including athlete wellbeing, academic / sport load, physical development, injury prevalence, psychosocial (e.g., life skill development), psychological (e.g., dual motivation) and educational factors (e.g., academic attainment) during athletes time at a sport-friendly school [29]. Whilst Thompson et al [28] offer an initial insight into the impacts experienced by athletes, these were based on the retrospective accounts of a subsample of all the student-athletes and staff within the sport-friendly school context. To progress our understanding, a more in-depth and in-time exploration of the various impacts and insights across a wider group of student-athletes is warranted. Furthermore, given the wide range of factors that need to be considered, a comprehensive assessment of holistic impacts and experiences within a sport-friendly school is likely to require the use of a variety of measurement tools and methods. Therefore, in order to explore what is it like to be a sport school student-athlete, this study aimed to evaluate the ’in-time’ multidimensional student-athlete holistic impacts and experiences from one sport-friendly school using a mixed-method research design.

## 2. Methods

### 2.1 Research approach

Evaluation research principles guided the methodological choices for this study [33]. In particular, Pawson and Tilley [34]’s realistic evaluation principles for an explorative case study approach. Critical realism informs both natural and social sciences but rebalances our focus back onto being rather than knowing, without losing sight of epistemological matters [35]. Although realistic evaluation often has the end goal of establishing a program theory (i.e., formulate preliminary explanations as to how impacts are produced in this context [34]) a first step is often to offer a descriptive account of what happens in terms of impact and experiences. In line with this premise, this study focused on an existing sport-friendly school program, that had not been evaluated for its holistic impacts and experiences on student-athletes before, with only very little theories explaining what outcomes may be expected and why or how these may emerge. The study, through the exploration of the emerging holistic impact patterns, predominantly aimed to provide an overview of the overall range of potential program impacts and experiences.

The study employed a mixed-methods triangulation design where the researchers used qualitative and quantitate methodology at the same time, to investigate holistic athlete development [36]. The qualitative and quantitative data were analysed separately but then integrated to triangulate findings; [37, 38]. Through triangulation, we attempted to cross-validate findings within a single study [39]. For example, the Family and free time KIDSCREEN-27 Health Questionnaire was used to measure student-athletes’ family and free time objectively and supportive open-ended questions to investigate a deeper and more contextualised description of family and free time impacts. Integrating quantitative and qualitative data can enhance the value of mixed methods research [39, 40]. Furthermore, mixed method designs are widely adopted in evaluation research [33, 41] and fits within the critical realist perspective [e.g., 42].

### 2.2 Context of study

One UK sport-friendly school (pseudonym–‘Eden High’) was selected for the study based on the Morris et al. [27] definition of a sport-friendly school. The selection of ‘Eden High’ was information-oriented and opportunistic. ‘Eden High’ has eight years of experience providing DC support through a performance sport pathway embedded within a UK independent school. Therefore, this school was an established and mature environment providing a rich source of information. ‘Eden High’ has eight performance sports as part of its performance programmes: athletics, basketball, cricket, football, hockey, netball, rugby and swimming, targeted at year groups 7–13 (aged 12–18 years). Each student-athlete who is a part of the performance programme follows a sport-specific scheme of work (e.g., physical, tactical and technical training). In addition, all of the student-athletes are enrolled within the standard school faculty. The flexibility of the curriculum allows them to combine their sporting programme with a range of options in General Certificate of Secondary Education (GCSE), Advanced level qualifications (A-level) and Business and Technology Education Council (BTEC) courses. As part of this program, ‘Eden High’ provides a study support programme, which includes: extra support and tutoring from teachers out of lessons, subject clinics, designated revision sessions, one-to-one meetings with teachers, academic mentors, learning development support and universities and colleges admissions service (UCAS) support. Overall, student-athletes enrolled on ‘Eden High’ performance sports program receive a place to study, train and sometimes live during their secondary school years, including access to learning facilities, a sports science centre, a sports treatment centre, sports facilities, accommodation buildings and a canteen all in one proximity (single campus). Based on the information above, we envisioned that the student-athletes embedded within ‘Eden High’ would offer information-rich experiences and the environment would be an information-rich context, widening the understanding of what it is like to be a sport-friendly school student-athlete.

### 2.3 Positionality of the researcher

Three days a week, the primary researcher (first author) worked within ‘Eden High’ as the lead strength and conditioning (S&C) coach. Therefore, the primary researcher was embedded within the context being studied. Furthermore, the primary researcher was previously a competitive student-athlete at a different sport-friendly school. As such, her applied experience of working within a sport-friendly school environment and her experience of being a sport-school student-athlete will have inevitably shaped her preconceptions. It may have influenced the study’s initial framing, design and analysis. The primary researcher has a unique understanding of sport-friendly school environments (e.g., the impacts of balancing boarding, sport and educational demands, the support services offered and the multiple interactions student-athletes have with other stakeholders within a sport-friendly school performance programme). These first-hand experiences of sport-friendly school environments allow the researcher to have a contextually relevant understanding of the various stakeholders’ language, behaviours and viewpoints, resulting in a more-depth evaluation of what it is like to be a sport-friendly school student-athlete [43].

### 2.4 Participants

Eighty-three year 12–13 student-athletes (Mage = 17.40 ± 0.53 years, male = 57, female = 26) from ‘Eden High’ participated in the study. Participants had to meet the following inclusion criteria: participate as an athlete in one of the performance sports programmes within ‘Eden High’ and be aged 16 or above. Overall, out of the 83 student-athletes, 36 were boarders and 47 were non-boarders, representing the following sports: athletics (n = 4), swimming (n = 1), cricket (n = 4), hockey (n = 13), netball (n = 9), football (n = 22), rugby (n = 18) and basketball (n = 12). Out of the 83 student-athletes 45% also participated in sport external to the school context. The student-athletes had been attending and competing at ‘Eden High’ for an average of 1.3 ± 1.4 years (range from 3 months to 7 years). As such, the sample included participants who had first-hand practical experience of being embedded within a sport-friendly school performance sports programme and the corresponding integrated educational institute and participants transitioning into the sport-friendly school environment.

### 2.5 Study design

Overall, in order to gain a comprehensive, holistic picture of what it is like to be a student-athlete at ‘Eden High’ and the impacts associated with the programme, five data collection methods were utilised: 1) online questionnaire, 2) physical fitness testing battery, 3) academic assessments grades, 4) injury data and 5) log diaries. The data collection methods were explicitly selected or designed to target key concerns regarding holistic athlete impacts outlined in the literature [28, 29]. Table 1 presents an overview of how each data collection method is mapped to identify holistic impacts and experiences of ‘Eden High’. This study was granted by Leeds Beckett University sub-ethics committee (Ref. 86728) with online informed participant assent collected prior to data collection and parental written consent obtained retrospectively.

**Table 1:** Instrument development joint display for triangulation purpose.

Category	Quantitative measurements	Qualitative measurements
Academic and sport workload	• Non-standardised questionnaire that captured student-athletes external sport, internal sport, extra-curricular and academic workload and difficulty balancing academic work and sport	• Supportive open-ended question, where student-athletes were able to provide rational and expand on the reason for their quantitative answer to the difficulty balancing academic and sport workload. • Log diary
Academic support	• Non-standardised questionnaire that captures the student-athletes satisfaction with academic support.	• Supportive open-ended question, where student-athletes were able to provide rational and expand on the reason for their quantitative answer to their satisfaction with academic support. • Log diary
School academic success	• School termly academic assessment grade	• Log diary
Sport Development	• Physical Development: Physical fitness tests (5-40m sprints, 505, CMJ, 30:15, MTP, anthropometric measures) • Technical/tactical Development:○ Athlete Sport Competence Inventory (SCI; [44])	• Log diary
Rest and recovery	• Sport Recovery-Stress Questionnaire (RESTQ-52-Sport; [45])	• Log diary
Injury & Illness	• Injury tracking database from Physio • Non-standardised questionnaire that captures injury and illness incidence, training sessions missed/adapted due to injury/illness, academic lessons missed due to injury/illness, and days off school due to illness.	• Supportive open-ended question, where student-athletes were able to provide how the school dealt, adapted and supported their injury • Log diary
Body image	• Eating Attitudes Test (EAT-26; [46])	• Log diary
Family and free time	• Family and Free Time KIDSCREEN-27 Health Questionnaire for Children and Young People [47]	• Supportive open-ended question, where student-athletes were able to provide rational and expand on the reason for their quantitative answers to the Family and free KIDSCREEN-27 Health Questionnaire • Log diary
Friends	• Friends KIDSCREEN-27 Health Questionnaire for Children and Young People [47]	• Supportive open-ended question, where student-athletes were able to provide rational and expand on the reason for their quantitative answer to the Friends KIDSCREEN-27 Health Questionnaire • Log diary
Life Skills	• Life Skills Scale for Sport (LSSS [48])	• Log diary
Sport Confidence	• Revised Competitive State Anxiety-2 CSAI-2R; [49])	• Log diary
Dual Career Motivation	• The European Student-Athletes’ Motivation Towards Sports and Academics Questionnaire (SAMSAQ-EU; [50, 51])	• Log diary
Resilience	• The Brief Resilience Scale (BRS; [52])	• Log diary

### 2.6 Data collection

All data was collected across a two-month period of the second half of the first term of a school academic year.

#### 2.6.1 Questionnaire

Data collection involved participants completing one online questionnaire that provided a multidimensional assessment of holistic athlete impacts and experiences—the questionnaire comprised of standardised and non-standardised questionnaires.

*2*.*6*.*1*.*1 Academic and sports workload*. A survey on athletes’ sport, academic and extra-curricular workload was developed (informed by [53, 54]) to capture external sport, internal sport, extra-curricular and academic workload and the difficulty balancing academic work and sport. As part of this survey, in order to assess workload athletes were asked on a slider scale to quantify over the last two months on average a week: 1) hours doing extra-curricular activities, 2) hours spent in academic lessons, 3) hours doing home-work, 4) number of academic lessons missed due to sport, 5) hours of external training hours, 6) number of external competitions, 7) number of external S&C sessions, 8) hours of internal training hours, 9) number of internal competitions, 10) number of internal S&C sessions, 11) number of performance analysis sessions, 12) number of recovery/rehab sessions and 13) number of rest days. For some of the measurements (e.g., competition), a broader measurement was taken (e.g., number of, rather than hours), as time is not standardised (e.g., due to different sports having different lengths of competition and not everyone playing full matches). In order to assess the difficulty balancing academic and sport workload the student-athlete were asked to rate on a five-point Likert scale (ranging from extremely difficult to extremely easy) how difficult they were currently finding balancing the academic work and sport. To further support this, there was an open-ended text box, where student-athletes were able to provide a rationale and expand on the reason for their answer to how difficult they were finding balancing academic and sport workload.

*2*.*6*.*1*.*2 Academic support and satisfaction*. Questions on academic support were developed to capture the student-athletes satisfaction with academic support. Student-athletes were asked to rate on a five-point Likert scale (ranging from extremely dissatisfied to extremely satisfied) how satisfied they were with the academic support they were currently receiving (one item). To further support this, there was an open-ended text box, where student-athletes were able to provide a rationale and expand on the reason for their answer to how satisfied they were with the academic support.

*2*.*6*.*1*.*3 Injury and illness*. A survey on athletes’ injuries and illness was developed to capture injury and illness incidence and their consequences (e.g., number of training sessions missed/adapted due to injury/illness). As part of this survey, student-athletes were asked in the two months prior to the questionnaire, regardless of the consequences for participation in regular training and/or competition, 1) if they had sustained an injury (yes/no), 2) state what injury they had sustained (open ended text entry), 3) how ‘Eden High’ had dealt, adapted and supported them with the injury (open ended text entry), 4) how many training sessions they had missed or had adapted due to their injury (slide scale) and 5) how may academic lessons they had missed due to their injury (slide scale). The same questions were then repeated but with regards to illness (not injury). Injury was defined as "*a physical complaint or observable damage to body tissue produced by the transfer of energy experienced or sustained by an athlete during participation in Athletics training or competition*, *regardless of whether it received medical attention or its consequences with respect to impairments in connection with competition or training*." [55 p. 2]. Illness was defined as “*a physical or psychological complaint or manifestation by an athlete not related to injury*, *regardless of whether it received medical attention or its consequences with respect to impairments*” [55 p.3].

*2*.*6*.*1*.*4 Rest and recovery*. The perceived level of stressors and recovery activities was assessed with the Sport Recovery-Stress Questionnaire (RESTQ-52-Sport; [45]). The RESTQ-52 sport has good construct validity and good-to-satisfactory internal reliability [45]. In addition, previous research has shown suitability for use with youth populations (e.g. [56, 57]). Participants were asked to respond to the items on a seven-point Likert-type scale anchored by descriptors ranging from “Never” (0) to “Always” (6), indicating how much the statements applied to them over the last eight-weeks. Nineteen subscale scores were derived, which were further grouped into the following four major subscale groups: 1) General Stress Subscale (General Stress, Emotional Stress, Social Stress, Conflicts/Pressure, Fatigue, Lack of Energy and Physical Complaints), 2) General Recovery Activity Subscale (Success, Social Recovery, Physical Recovery, General Well-being and Sleep Quality), 3) Sport-specific Stress Subscale (Disturbed Breaks, Burnout/Emotional Exhaustion and Injury) and 4) Sport-specific Recovery Activity Subscale (Being in Shape, Personal Accomplishments, Self-efficacy and Self-regulation).

*2*.*6*.*1*.*5 Body image*. The Eating Attitudes Test (EAT-26; [46]) was used to assess at-risk eating behaviour [58]. The 26-item version is highly reliable and valid [59, 60] and has been used previously to assess “eating disorder risk” in sports schools, high schools, colleges and other unique risk samples such as athletes [61, 62]. Items are presented in a 6-point Likert scale ranging from 1 ("never") to 6 ("always). Increasing EAT scores seem to be indicative of increased eating pathology [60]. Taking several studies into account, a cut-off score of 10 is a sufficiently distinct measure of subthreshold cases [63, 64]. Hence, in the current study, subjects scoring EAT 0–9 are defined as subjects with normal eating behaviour attitudes and EAT ≥ 10 as subjects with disordered eating behaviour and attitude.

*2*.*6*.*1*.*6 Family*, *free time and friends*. The autonomy and parent and peer and social support sub-scales from the KIDSCREEN-27 Health Questionnaire for Children and Young People [47] were used to evaluate participants’ relationships with parents, feelings of having enough age-appropriate freedom and the nature of the respondents’ relationships with other children/adolescents. For this study, the degree of satisfaction with financial resources was removed. For the peer and social support sub-scales scores were converted to Rasch person parameter estimates and then into T-values, using the SPSS syntax provided resulting in subscale scores with a scale mean around 50 and standard deviation around 10, with higher values indicating, higher HRQoL [47]. The items within the KIDSCREEN-27 questionnaire are suitable for children and adolescents aged 8–18 years [65]. All domains have reported satisfactory-to-good internal consistency and a two-week test- reliability was deemed adequate for all dimensions [65]. Participants were asked to answer items regarding the last eight-weeks on a 5-point Likert-type scale (never, seldom, quite often, very often, always). In addition, an open-ended question was added where student-athletes could elaborate on how the features of ‘Eden High’ had influenced their answers to the KIDSCREEN-27 Health Questionnaire sub-scales. Open-ended questions have been used in previous research to help expand on responses to close-ended questions [66].

*2*.*6*.*1*.*7 Sport competence*. Athletes’ perceived competence in sport was measured using the Sport Competence Inventory (SCI; [44]). The SCI measures athletes’ self-perceptions of their sports competence using three items that assess technical, tactical and physical skills. The student-athletes rated their competence in these areas on a 5-point scale ranging from "not at all competent" to "extremely competent". The questionnaire has previously demonstrated adequate psychometric properties, reliability and validity, in addition to being suitable for use within youth populations [44].

*2*.*6*.*1*.*8 Sport confidence*. The self-confidence subscale of the Revised Competitive State Anxiety-2 (CSAI-2R; [49]) was used to assess athletes’ confidence in sport. This measure is composed of five items rated on a 4-point scale ranging from "not at all" to "very much so". Previous research has shown suitability for use with youth populations [e.g., 67] and established good factorial validity [49].

*2*.*6*.*1*.*9 Life skills*. The 43-item Life Skills Scale for Sport (LSSS [48]) measured participants’ perceived life skills development through sport. Participants were asked to "rate how much you perform the skills listed below." Responses were provided on a 5-point scale ranging from 1 (Not at all) to 5 (Very much). Example items included: teamwork (7 items; ’’work well within a team/group’’), goal setting (7 items; ’’set specific goals’’), time management (4 items; ’’manage my time well’’), emotional skills (4 items; ’’use my emotions to stay focused’’), interpersonal communication (4 items; ‘‘communicate well with others”), social skills (5 items; ‘‘interact in various social settings”), leadership (8 items; ‘‘organise team/ group members to work together”) and problem solving and decision making (4 items; ‘‘think carefully about a problem”). Previous research provided evidence for the validity and reliability of this scale with youth sports participants [48].

*2*.*6*.*1*.*10 Motivation toward a dual career*. The European Student-Athletes’ Motivation Towards Sports and Academics Questionnaire (SAMSAQ-EU; [50, 51] was used to establish the student-athletes motivation for a dual career [68]. Participants were required to indicate their level of agreement (i.e., from a minimum of 1 –very strongly disagree to a maximum of 6 –very strongly agree) to each SAMSAQ-EU item [50, 69]. The questionnaire has been shown to being suitable for use within youth populations [e.g., 70].

*2*.*6*.*1*.*11 Resilience*. The Brief Resilience Scale (BRS; [52]) was used to assess participants’ ability to bounce back from stress (resilience). The BRS has shown high test-retest reliability and validity [52], in addition to being suitable for use within youth populations [e.g., 71]. The scale consists of five items that measure the ability to recover after stress rated on a 5‐point Likert scale (1 = strongly disagree; 5 = strongly agree). The BRS is scored by calculating a mean of the six items.

#### 2.6.2 Physical fitness testing battery

In order to assess physical development, a fitness testing battery which included; lower-body power, strength, speed and cardiovascular fitness tests was conducted in line with previous studies [72]. Speed was reported at 10- and 40-m distances [73], cardiovascular fitness was reported based on the 30–15 intermittent fitness test (IFT) [74], lower-body power was reported using counter-movement jump (CMJ) height (m) and strength was reported using the isometric midthigh pull (IMTP) [75] peak force (kg) and relative peak force (kg^-1^) measures. In line with previous studies [e.g., 72, 76], the IMTP was performed using a Wooden Custom Isometric midthigh pull rig, the CMJ was performed using the Optojump system (Microgate, Bolzano, Italy), linear sprint speed was assessed using single beam timing gates (Whitty timing gates) and 30-15IFT following the protocol outlined by Buchheit [74]. The fitness testing battery was conducted over two weeks. In week one, subjects performed measures of strength via the IMTP and power via the CMJ. On week 2, field-based measures of 10–40 m sprints to measure speed and 30-15IFT to measure cardiovascular fitness were performed. On all testing days, the test causing the greatest strain on the neuromuscular system was performed first to enhance the reliability of all maximal testing procedures [77].

#### 2.6.3 Academic assessments grades

To assess educational attainment, end-of-term academic subject assessment grades were extracted from the school administrative system. As all student-athletes were in years 12–13, grades were provided in the UK national curriculum grading format for A-level and BTEC qualifications. To adequately compare BTEC and A-level grades, in addition to statistical purposes, academic assessment grades were converted to a number using a school grades translation matrix in Table 2 (similar to Sports Council, 1993). After conversion, an average of each individual subject score to get one overall academic assessment score for each student-athletes.

**Table 2:** School grades translation matrix.

A-Level Assessment Grade	BTEC Assessment Grade	Awarded Score
A*	D*	6
A	Di	5
B	M	4
C	P	3
D	-	2
E	-	1
F	F	0

#### 2.6.4 Injury data

In order to assess injury incidence and the types of injuries sustained, the school’s physio practitioner provided a log of the number, type and anatomical site of injury over two months in the academic year (aligned with period of data collection for the questionnaire).

#### 2.6.5 Log diary

Student-athletes were asked to fill in a singular log diary comprised of open-ended questions that explored the positive and negative impacts on student-athletes holistic (athletic/physical, academic, psychosocial and psychological) development and any factors that caused, attributed, or drove these impacts/outcomes. Open-ended questions allowed the respondents to express an opinion without being influenced by the researcher [78]. For example, student-athletes were asked to reflect on the last month and outline the positive and negative impacts they had experienced on their academic/vocational development. Furthermore, open-ended questions allowed respondents to include more contextual information, providing more feedback on sport-friendly school student-athlete holistic impacts [66]. For example, student-athletes were asked to explore what caused, attributed to impacts/outcomes happening.

### 2.7 Data analysis

Due to the mixed method approach, a triangulation design (as per 2.1) was used whereby quantitative and qualitative data were firstly analysed separately. Then, given that qualitative and quantitative findings about impacts and experiences are complementary allowing for triangulation, the findings were integrated and presented together.

#### 2.7.1 Quantitative analysis

All quantitative statistical data were processed using SPSS (V26., IBM) and Excel (Microsoft Office 2021). Results are described using either means with SD, medians with ranges or frequencies with percentages. Scale reliability for the standardised questionnaires was assessed using Cronbach’s alpha. Table 3 shows the summary of the standard questionnaires scale reliability. Convention stipulates that an ɑ < 0.50, signifies poor reliability and ɑ > 0.70 signifies good reliability [79]. Low alphas can be caused by scales consisting of few items. In this case, scales can still be usefully included in the analysis [79], as long as the low alpha value is discussed as a limitation [80]. The SCI (competence) scale and BRS comprises only three and five items which may explain its low alpha. With this in mind, no items were removed from the analysis, although caution may be warranted in interpreting the SCI (competence) and BRS subscales. SAMSAQ-EU questionnaire was calculated based on Lupo et al. [51] federation three-factor model (SAM = 12 items; AM = 13 items; and CAM = 5 items), as this represented student-athletes classified according to the UK dual career policy.

**Table 3:** Standard questionnaires scale reliability.

Questionnaire	Cronbach’s Alpha	Cronbach’s Alpha Based on Standardised Items	Number of Items
SCI (competence)	0.631	0.625	3
Revised CSAI-2R	0.909	0.911	5
SAMSAQ-EU	0.831	0.863	28
RESTQ-52	0.808	0.806	53
EAT-26	0.681	0.723	25
BRS	0.562	0.558	6
Kidscreen-27 (Family and Free Time)	0.733	0.732	5
Kidscreen-27 (Friends)	0.841	0.847	4
LSSS	0.942	0.944	43

Note: Cronbach’s alpha employs the covariances among the items, Cronbach’s alpha based on standardised items employs the correlations among items.

#### 2.7.2 Qualitative analysis

This study aimed to provide an exploratory (i.e., seeking to describe a phenomenon) approach to research [81]. As such a qualitative content analysis approach was adopted to generate descriptive themes. The purpose of content analysis is to describe the characteristics of the finding’s content by examining who says what, to whom and with what effect [82]. Within this study Elo and Kyngäs [83] phases to content analysis were followed. Firstly, during the preparation phase, qualitative data was organised and managed into categories, in order to be analysed together (i.e., log-diaries and open-ended questionnaire data) and the primary researcher obtained a sense of the whole data through reading the transcripts several times. Next, during the organisational phase, data was generated through coding [84]. Our coding approach was deductive in so far as most codes were generated through the research team’s prior literature review [29] and targeted key concerns regarding holistic athlete impacts and experiences (refer to Table 1). Inductive coding was used as new themes specific to holistic impacts of student-athletes were identified during the coding process. Once the primary author coded the data, the four authors met to discuss the categories identified and their understanding and interpretation of the data. The themes were further refined during this process and were explored in a way that would allow the research question to be addressed. For example, life skills and resilience were grouped into inter and intra personal skills.

#### 2.7.3 Triangulation

Given that qualitative and quantitative methodology were chosen to investigate the same key concerns regarding holistic athlete impacts and experiences outlined in the literature they subsequently complement and triangulate with each other [85]. As such, although the data were extracted from various methods, the data for analysis were compatible for integration using the process of triangulation [86]. As part of this process, the primary researcher compared the findings from each component of the study and considered where the finding from each method agree (converge), offer complementary information on the same issue (complementarity) or appear to contrast each other (discrepancy or dissonance) [86]. Subsequently, the assessment of convergency, complementary and discrepancy were discussed among the four researchers to (a) clarify interpretations of the findings and (b) determine degree of agreement among researchers on triangulated findings [86].

## 3. Results

In the results section, we present the integrated findings of the ’in-time’ multidimensional and varied holistic impacts and experiences of sports school student-athletes from one sport-friendly school.

### 3.1 Combining of sport and education

#### 3.1.1 Sport and academic workload

Student-athletes academic and sport workload per week included 26.7 ± 9.9 hours of academic work (consisting of 19.1 ± 7.0 hours of academic lessons and 7.5 ± 5.7 hours of homework), alongside 10.5 ± 4.4 hours of sports training (including technical and tactical sessions), two (range from 0–6) competitions, two (range from 1–6) S&C sessions, one (range 0–3) rest day, one (range 0–3) recovery/rehab session, performance analysis (range from 0–5 sessions) and nutrition workshops. This high sport workload resulted in student-athletes at ‘Eden High’ missing on average one (range 0–12) academic lesson a week.

Student-athletes who balanced education, school sport and out-of-school sport (55%) undertook, on average, an extra 2.8 hours of training, two competitions and one S&C session a week compared to student-athletes who balanced solely education and school sport.

#### 3.1.2 Sport and academic experience

Table 4 shows the mixed-method findings of student-athletes’ experiences of combining and balancing academics and sport in a sport friendly school. Fifty-one percent of student-athletes found it neither easy nor difficult to combine and balance their academic work and sport. Twenty-nine percent, 8%, 11% and 2% found it somewhat difficult, extremely difficult, somewhat easy and extremely easy, respectively.

**Table 4:** Mixed-method findings of the experiences of students combining and balancing academics and sport.

	Number of Student-Athletes	Percentage of Student-Athletes	Reasons Why
**Extremely difficult**	5	8%	• Feeling overwhelmed with balancing sport load and academic work • Extra workload due to exams • Lack of time • Missing a lot of school
**Somewhat difficult**	19	29%	• Desire to commit more to sport • Lack of time due to balancing university applications, weekly/frequent tests, exams/revision, rest/recovery and a heavy fixture schedule all at once • Missing lessons due to sport • Academic pressure (e.g., due to scholarship and aspiring career path i.e., medic) • Tiredness • Large travel time for day pupils
**Neither easy nor difficult**	33	51%	• Have sufficient time within school and outside school to manage work • Manage time effectively (i.e., time management, being organised and planning their week) • Ability to cope with the workload (e.g., learnt to deal with the pressures) • A balance between sport and academics workload • Didn’t have exams at time • Utilised study time effectively (e.g., study sessions instead of training when needed, prep/night home sessions, utilised free periods in school day, utilised weekends to work when necessary)
**Somewhat easy**	7	11%	• Sufficient time for work • Know when to prioritise work or sport depending on week • Get all work done in school hours • Don’t fall behind
**Extremely easy**	1	2%	/

Summarising the qualitative findings of the online questionnaire, the student-athletes who found it easier to balance sport and academic workload generally attributed this to: managing their time effectively, dealing/coping with the pressures, having a balance between sport and academic workload and utilising their study time effectively. In comparison, those athletes who found it harder to balance academic and sports workload felt this was due to a desire to commit more to sport, missing lessons due to sport, academic pressures, lack of ability to cope with the workload, tiredness, considerable travel time due to being a day pupil and a lack of time (e.g., due to university applications, exams, rest and fixtures).

#### 3.1.3 Dual career motivation

Overall, at ‘Eden High’, based on the SAMSAQ-EU questionnaire, student-athlete’s academic motivation (motivation toward academic-related tasks) was 4.85 (± 0.88), student athletic motivation (motivation toward elite sport) was 4.43 (±0.83) and career athletic motivation (motivation to pursue a professional sports career) was 2.63 (±0.71). As a result, student-athlete at ‘Eden High’ demonstrate equal motivation toward academics and elite sports; however lower motivation to pursue a professional sports career.

### 3.2 Academic

#### 3.2.1 Academic assessment grades

Table 5 shows the summary of the student-athlete academic assessment grades. Majority (95%) of student-athletes achieved an academic assessment grade of four or higher, signifying a B (A-level), merit (BETC) or above in the UK system. Only 5% (n = 3) of student-athletes obtained a three academic assessment grade and no student-athlete obtained a two or lower grade.

**Table 5:** Summary of academic assessment grades.

Academic Assessment Grade	Number of Student-Athletes	Percentage Student-Athletes
6	3	5%
5	27	42%
4	31	48%
3	3	5%
2	0	0%
1	0	0%
0	0	0%

#### 3.2.2 Satisfaction with academic support

Table 6 shows the student-athletes satisfaction with academic support at ‘Eden High’. 67% of student-athletes had a positive-valence towards academic support, 11% a negative valence and 22% a neutral valence.

**Table 6:** Mixed-method findings of the student-athletes satisfaction with the academic support.

	Number of Student-Athletes	Percentage of Student-Athletes	Reasons Why
**Extremely dissatisfied**	1	2%	/
**Somewhat dissatisfied**	6	9%	• Poor teaching • Timings of academic support sessions (e.g., not convenient/optimal)
**Neither satisfied nor dissatisfied**	14	22%	• Timings of academic support sessions (e.g., not convenient/optimal) • Lack of encouragement from teachers to attend clinics • Received academic support but took too long to be initiated
**Somewhat satisfied**	31	48%	• Inconsistency in the support student-athletes received dependent on time of year, teacher and subject • Careers department • Consistent check-ups from academic and sport staff
**Extremely satisfied**	12	19%	• Always have someone to go too/always received help when needed • Good teacher support • Extra revision sessions • Academic support available during school as well as after school

Majority of student-athletes experienced an environment where they received good academic support when needed (e.g., teacher support, extra revision sessions, future career support) and consistent academic and sports staff check-ups. However, some student-athletes stated that the academic support were not always convenient (e.g., after school/late), particularly for day students; as exemplified by athlete 1, *"the clinics are usually very late*, *which is not useful for day students*.*"* Furthermore, that the student-athletes experienced inconsistency in the support they received and sometimes the academic support was not advertised or pushed by staff, as testified by athlete 2: *“the support is there when needed but not advertised or consistent as it could be*.*"*

### 3.3 Athletic and physical

#### 3.3.1 Sport competence, development and confidence

Table 7 shows the findings in relation to student-athletes’ sports competence and enhanced performance. Student-athletes rated their sports competence between moderate to very competent (3.56 ± 0.81). Technical competence was rated the highest, then tactical, then physical. From the sport confidence inventory, the student-athletes reported moderate sport confidence scores, 2.81 (± 0.67). Student-athletes stated in their log diaries that they had developed sporting confidence, as demonstrated by athlete 18: *“I have gained confidence in my ability and have been more motivated to improve*.*”*

**Table 7:** Mixed-method findings of student-athletes sport competence and enhanced performance.

	Sport Competence Score (a.u.)	Reasons Why
**Technical**	3.66 ± 0.80	• High-standard competition providing challenging situation to practice and develop key skills • High standards/expectation from coaches • High quality training partners • Positive team culture (coaches and players supporting, pushing and encouraging each other)
**Tactical**	3.60 ± 0.84	• Video analysis
**Physical**	3.43 ± 0.77	• Sport science support (e.g., S&C, physio, nutrition)

Note: 5-point Likert scale ranging from 1 (not at all competent) to 5 (extremely competent)

In the physical fitness testing battery, the average scores were as follows; 10m sprint 1.96 ± 0.54 seconds, 40m sprint 5.80 ± 0.54 seconds, CMJ 36.6 ± 7.6 cm, absolute MTP 136.0 ± 39.5 kg, relative IMTP 1.81 ± 0.37 kg^-1^ and 30–15 IFT 17.9 ± 2.1. Furthermore, the student-athletes stated in their log diaries that being a student-athlete at ‘Eden High’ improved and enhanced their physical and technical development and overall sporting performance. *“Improved physically within the gym*, *understood what I am doing wrong in my game through video analysis and started to implement things to improve my game" [Athlete 3]*. As a result, some student-athletes played at a higher level of sport than before. *"I have become better at fitness levels*. *I started to play at a higher level outside of school*. *I feel a lot better within myself physically and mentally” [Athlete 4]*.

When prompted to reflect on what drove their technical, tactical and physical development, the student-athletes stated; high standard of competition, high standards/ expectations from coaches, high-quality training partners, a supportive and positive team culture, video analysis and sport science support. For example, athlete 5, when asked in the log diary to explain what had caused, attributed or drove their athletic/physical impacts, responded, *“playing to a high standard as much as possible*, *with coaches and leaders in the team not letting standards drop*.*”* Furthermore, athlete 6 stated, “*Played with high standard players around me drive me on to do as well as I can and improves my play*. *We have had some good results and I have definitely improved*.*”* However, some athletes stated that the academic workload (e.g., exams) sometimes interfered with their physical training (e.g., missing training due to revision), causing student-athlete rehabilitation or physical development to take a back seat, potentially slowing down performance progress.

“Having so much work has meant I have missed a couple of sessions so that I could revise, but I wish I did not have to as I missed out on a session which could have been beneficial to me. When I do play my sport, it is a great release and a way to forget about all the school stress." [Athlete 7]

#### 3.3.2 Risk of injury and illness

**Fig 1:**
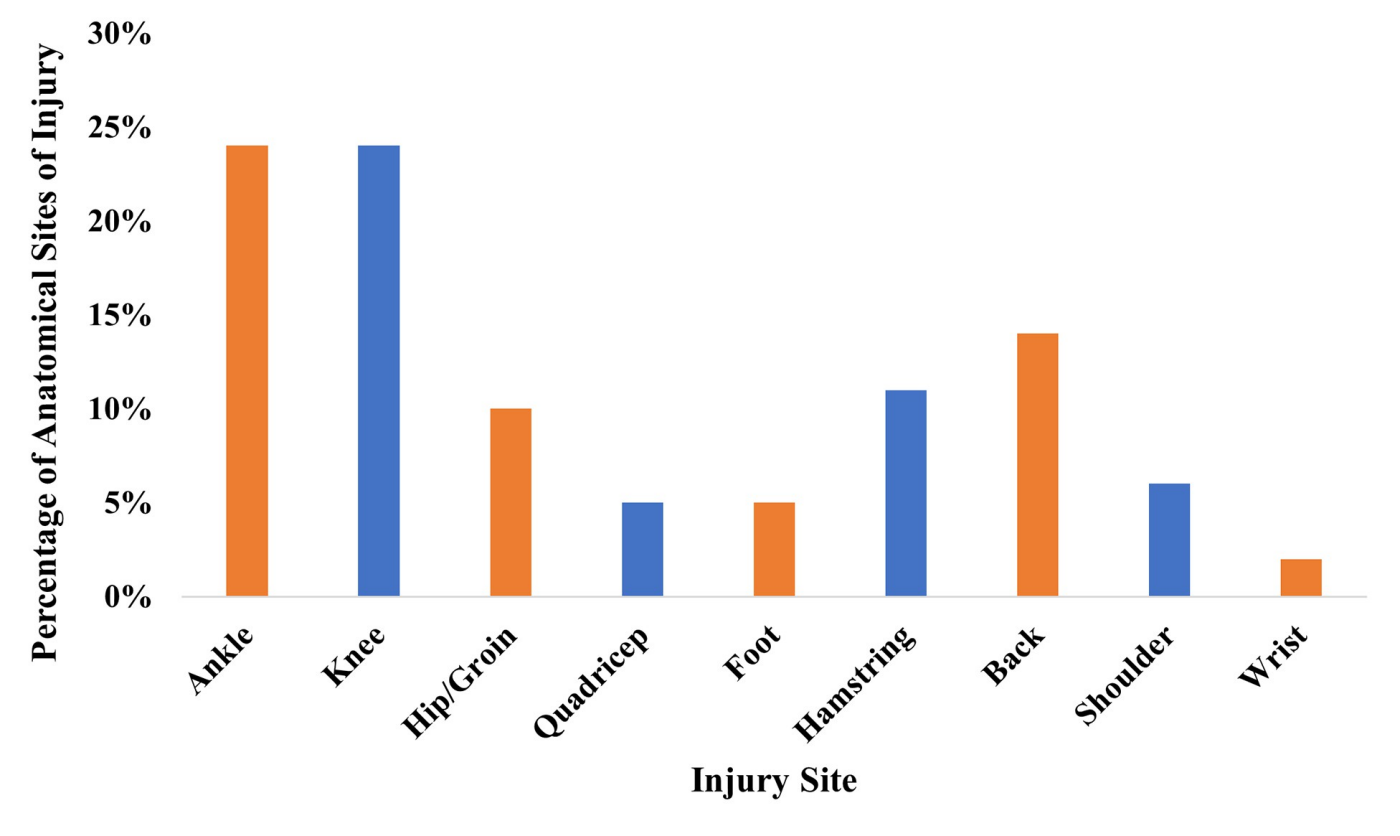
Summary of the anatomical sites of injury.

Forty-six per cent (n = 40) of student-athletes at ‘Eden High’ reported having had an injury and 20% (n = 13) illness over a ten-week period. According to the physio reports, the most common injury sites were ankle (24%) and knee (24%). Fig 1 summarises the anatomical sites of injury.

#### 3.3.3 Athletes experience of injury and illness

Overall, student-athletes missed/adapted five (range 0–40) training sessions and zero (range 0–12) academic lessons due to injury. Additionally, student-athletes missed one (range 0–6) training session, three (range 0–9) academic lessons and two (range 0–5) days off school due to illness. As such, student-athletes get injured and ill but only appear to impact sport and education in a minority of cases. The high number of training sessions missed due to injury were not common within the data set (outliers), however, it does highlight the individual nature and severity of injuries within the cohort.

The student-athletes highlighted that ‘Eden High’ had a physio practitioner and sports massage therapist available to them for two days a week. As one student-athlete noted: *“we receive constant support from the physio as well as sport massage*.” The physio assessed sports injuries and provide the student-athletes with physio programs to help with their rehab and return to play. However, although the student-athletes had access to a physio, they stated they experienced a lack of appointments. Furthermore, it was slow for some student-athletes to get seen by the physio because the physio was only available two days a week, as explained by athlete 10, *“very slow on getting physio appointments*, *but once they have been scheduled it’s fine*.*”* The student-athletes also highlighted an environment where S&C staff provided mobility, warm-up, recovery resources and stretching/mobility sessions to the student-athletes as necessary. In addition, the S&C were seen to adapt training programs as necessary around injuries and rehabilitation and players were given time of training by coaches when needed; as represented by athlete 11, *“adapted weights program by S&C staff has been a big help*.*”* This additional support is further endorsed by athlete 12, *“due to injury*, *it has been a challenge*, *but support from the football coaches*, *physio and the strength and conditioning team help me maintain a positive attitude throughout my recovery*.*”* Finally, student-athletes stated having access to ice baths and stretching bands to help aid their injury prevention and recovery.

#### 3.3.4 Rest and recovery

**Fig 2:**
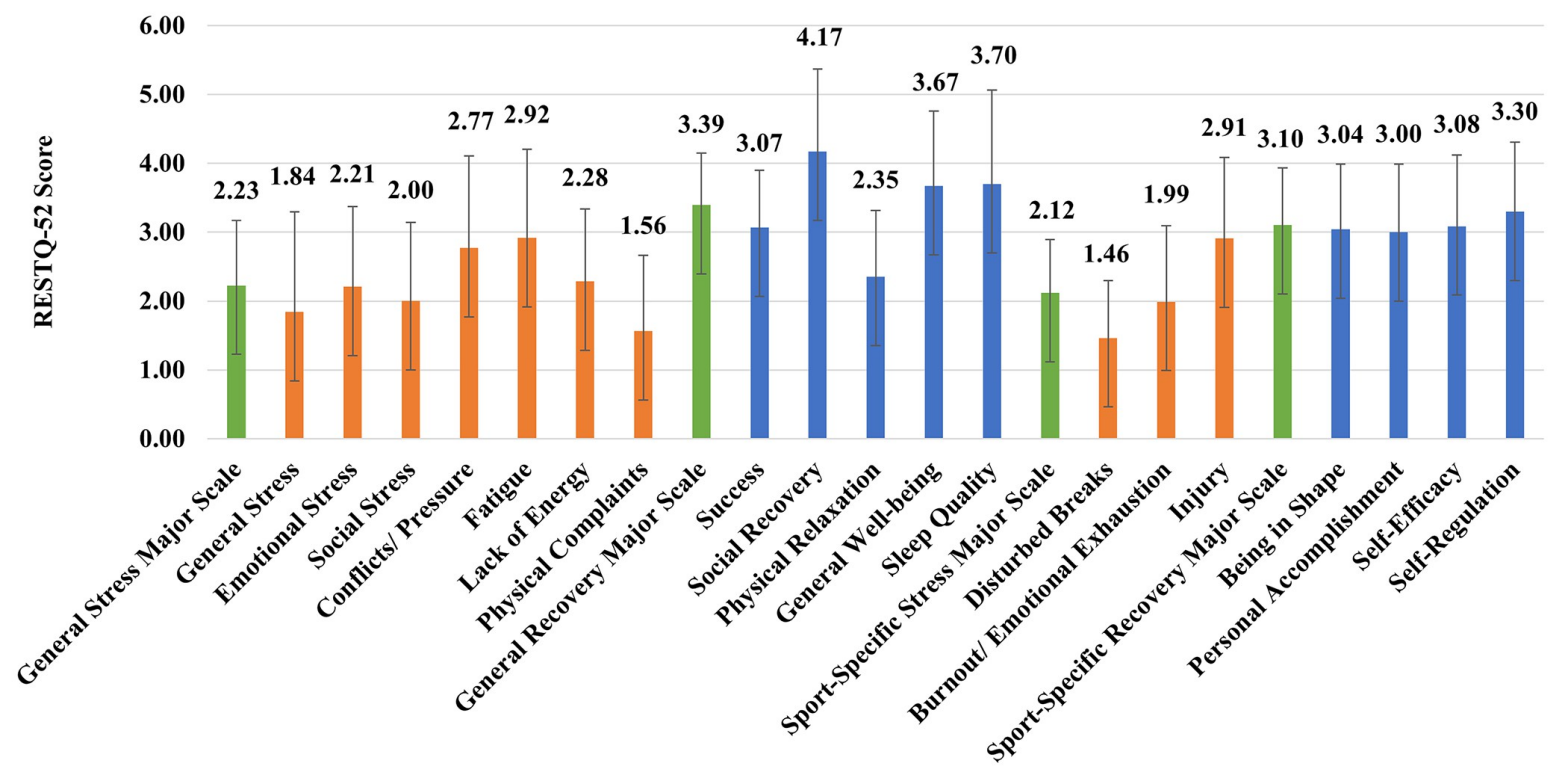
Summary of RESTQ-52 data. Note: Major subscale groups: 1) General Stress Subscale (General Stress, Emotional Stress, Social Stress, Conflicts/Pressure, Fatigue, Lack of Energy and Physical Complaints), 2) General Recovery Activity Subscale (Success, Social Recovery, Physical Recovery, General Well-being and Sleep Quality), 3) Sport-specific Stress Subscale (Disturbed Breaks, Burnout/Emotional Exhaustion and Injury) and 4) Sport-specific Recovery Activity Subscale (Being in Shape, Personal Accomplishments, Self-efficacy and Self-regulation).

A summary of RESTQ-52 data is displayed in Fig 2. Overall, the general stress major scale score was 2.23 ± 0.95, general recovery major scale was 3.39 ± 0.75, the sport-specific stress major scale was 2.12 ± 0.77 and the sport-specific recovery major scale was 3.10 ± 0.83. As such, RESTQ-52 data suggested student-athletes’ were only "sometimes" stressed and "often" recovered. The higher injury (2.91 ± 1.17) and lower physical relaxation scores (2.35 ± 0.96) are coherent with the injury rate highlighted above (section 3.3.2). Furthermore, the conflicts/pressure score (2.77 ± 1.33) is consistent with the challenge of combining sport and education (section 3.1.2).

When considering the qualitative data, findings are consistent with the RESTQ data for fatigue scale (2.92 ± 1.29) where student-athletes expressed in their log diary that the demands of combing both education and sport resulted in a number of student-athletes becoming *“fatigued”* (both mentally and physically) and de-motivated. This is supported by athlete’s 12 log diary entry, *“Academically—lots of stressful situations which have caused me to feel demotivated and has impacted my sport*. *This has tired me greatly and continues to make me feel fatigued*.*”*

### 3.4 Psychosocial

#### 3.4.1 Inter and intra personal skills

**Fig 3:**
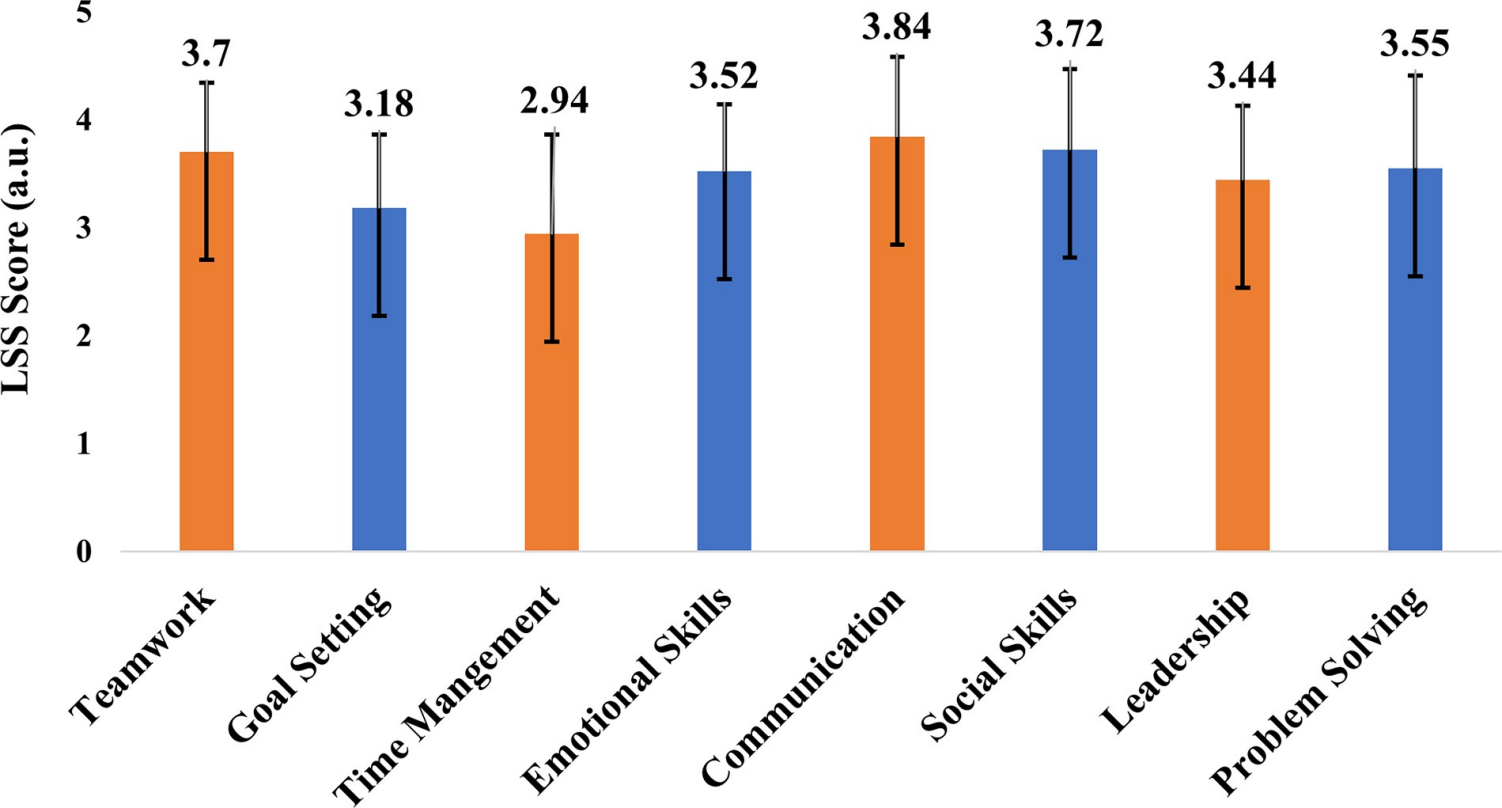
Breakdown of the LSSS scores.

Overall, the student-athletes average LSSS score was 3.49 ± 0.79 demonstrating they perform these life skills “sometimes” to “a lot”. Fig 3. shows the full breakdown of the LSSS Scores. The student-athletes scored lowest for time management 2.94 (± 0.92). Communication, social skills and teamwork were the highest LSS scores, which is supported by the qualitative data, as noted by athlete 13, *“I have increased in social skills due to opportunities in where I was able to meet new people*,*”* and athlete 13, *“I have become more sociable with my team and have gained confidence*,*” [athlete 13]*. Furthermore, throughout the log diaries, student-athletes stated developing several other life skills, such as enhanced responsibility and becoming role models.

From the BRS, the brief resilience score for the student-athlete at ‘Eden High’ was 3.49 (± 0.78).

#### 3.4.2 Social support and peers

The average score for the KIDSCREEN-27 social support and peers questionnaire was 15.57 (± 3.06) out of 20. The social support and peers T-value based on the Rasch person parameter (ages 12–18 years) was 46.2–49.4. The data demonstrates a feeling of acceptance, support and inclusion in peer groups. When combining this with the qualitative data, this result is unsurprising. It was evident from the log diaries and online questionnaire that the student-athletes felt part of a team, where they spent a significant amount of time together, interacting in class, training (up to seven times a week), competitions, living (e.g., boarding) and leisure. “*Because we play sport or are in the gym together*, *we spend a lot of time with each other"* [athlete 14]. These factors provided “*a platform for me to make new friends and is an environment for me to spend time with my friends*," as represented by athlete 15. These opportunities are further supported by athlete 16, *“we always spend a lot of time together*, *which allows us to create closer bonds*.*”* Additionally, student-athletes had further social support from coaches, teachers, house-parents and pastoral staff; as expressed in athlete 17’s log-diary, *"my peers and teacher have helped me relieve my stress with different methods such as yoga*.*”*

#### 3.4.3 Family and free time

The average KIDSCREEN-27 parent relations and autonomy questionnaire score was 17.5 ± 3.5 out of 25. Seventeen out of 25 seemed to indicate that student-athletes felt positive about the relationship with parents and having enough age-appropriate freedom to choose (e.g., things for yourself in the relationship). Conversely, despite student-athletes having a support network (e.g., team, sporting community, pastoral care) and increased opportunities to make and meet new friends, one consequence of balancing a high academic workload and sport was a lack of free time. Student-athletes had less time to socialise and contact time with friends and family within and outside of school and had to make extra-curricular sacrifices. At ‘Eden High’ only 14% (n = 9) of student-athletes did extra-curricular activities.

*"I find it difficult to find time to do things after school because after school I’ll have club or something*, *then diner at 5–6*, *then I have to do school work and I just can’t find time*.*"* [Athlete 15]

Forty-two per cent of student-athletes were boarders (lived on school site). As a result, they are away from home, family and friends outside of school (only get to see them on term holidays/weekends), limiting time spent with them. Some student-athletes described boarding in the online questionnaire as *“sheltering them from outside social experiences*.*”* Furthermore, boarding was stated to *“limit freedom”*, sometimes becoming a *“socially intense environment*”, where student-athletes were *“constantly surrounded by people”*, *“limiting time to yourself*.*”*

### 3.5 Psychological

#### 3.5.1 Mental health

Although being a student-athlete at ‘Eden High’ may be associated with more pressure, training demands and expectations, findings from the log diaries only highlight the important influence sport within the school may have on student-athletes mental health. As highlighted above (section 3.1), student-athletes at ‘Eden High’ spend considerable time engaging in sports/physical activity. Many of the student-athletes stated in their log diaries that having sport timetabled into their day gave them *“a break from academics”*, giving them a *“stress relief”*, allowing them to regenerate and recharge, as explained by athlete 15, “*being able to train and become better every day helps a lot for my mental health and provides a lot that I can do on and off the pitch*.*”* Furthermore, student-athletes in their log diaries stated they were *“mentally stronger”*, *“happier” and* had *“better mental health”* from being a student-athlete. However, not everyone felt the same balance, as demonstrated by athlete 16, *"I have been stressed recently*, *which has taken a toll mentally*. *I have also been quite frustrated and angry with my sport/team and injuries*.*"* Some student-athletes found the environment and balancing social life, sports and academics challenging, resulting in increased stress and mental and physical fatigue/tiredness.

*“I have a lot more on my plate now; I’m more stressed because of UCAS and personal statements and then when I get onto the court*, *I don’t perform as well because I am just stressing about outside of school problems and so compared to last month I will say I am a bit more stressed*.*”* [Athlete 17]

#### 3.5.2 Body image

The overall average EAT-26 score was 6.26 ± 5.38, with 83% (n = 54) scoring between 0–9 and 17% (n = 11) equal to or greater than 10, signifying subjects with disordered eating behaviour and attitude. As a result, body image is a concern for 17%, highlighting a potentially negative impact on student-athletes.

## 4. Discussion

**Fig 4:**
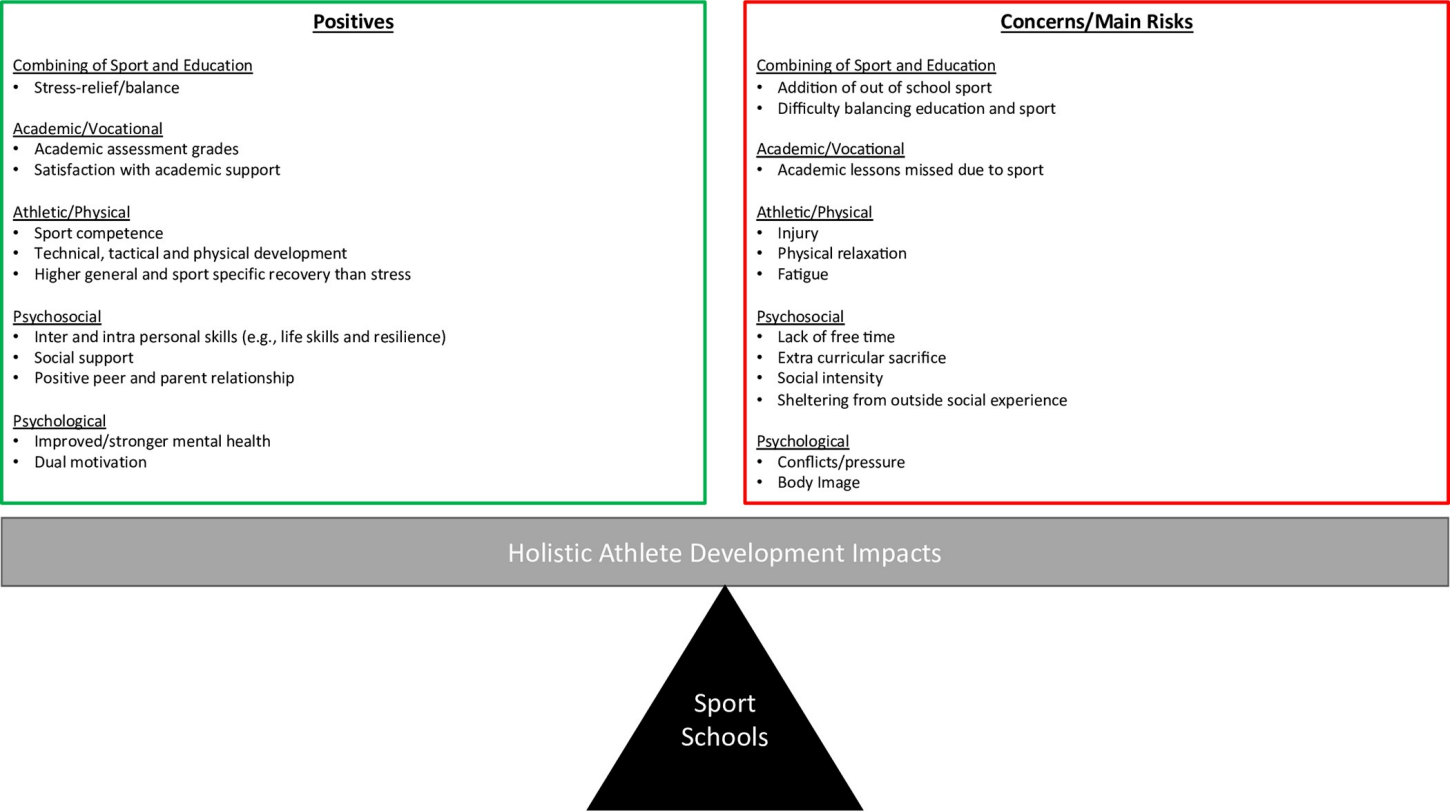
Summary of the positive and concerning holistic athlete impacts.

This study is the first to assess the ‘in-time’ impacts and experiences of sport-friendly school student-athletes across all four areas of holistic athletic development (i.e., educational/vocational, physical/athletic, psychosocial and psychological) using a mixed method multidimensional research design. Overall, the findings demonstrated there were a multitude of positive impacts and experiences associated with being a UK sports-friendly school student-athlete, but also impacts / experiences of concern (see Fig 4). Similar to recent consensus statements [4, 87] this study warns about the risk of several negative impacts associated with intensified youth sport programs. However, similar to Rongen et al. [1] our findings show that there are many potential positives impacts and experiences of youth sport programmes. Whilst the data has presented the mean or themed data, large standard deviations and ranges across the holistic impacts and experiences were apparent, which reflects the inter-individual variability in impacts and experiences of being a sport school student-athlete.

Previous research showed that student-athletes combined intense routines of sport and education [29]. However, this study was the first to quantify the academic and sport workload and the subsequent student-athlete experience. Overall, consistent with previous sports school research [e.g., 28], student-athletes had to balance a high physical and academic workload (e.g., 26.7 ± 9.9 hours of academic work alongside 10.5 ± 4.4 hours of sports training and two competitions a week). When comparing the data against age matched athletics (8.92 ± 3.69 h/wk [88]) and academy rugby league athletes (301 ± 92 minutes [89]) typical training volumes, the sport school students reported a higher physical workload. However, the sport and academic standard deviation and ranges were high, reflecting variation in terms of workload within sport-friendly school settings. The challenges of within- and between youth-athlete variance in weekly training load have been highlighted in previous research [e.g., 54, 90]. This variation could be explained by sport, representative level and school only vs. school and non-school sport [54]. Therefore, the individual needs of each student-athlete within a sport-friendly school will differ depending upon the sport, the academic pathway and their individual circumstances [91]. As such, individual antecedents and characteristics (e.g., sport, representative level) are likely to shape both the sport school experience and the impact it has on the holistic development of youth athletes. As such, future research should explore the interaction effect between characteristics and antecedents and the features and mechanisms that might result in the variation in impacts. Furthermore, to account for these individual differences flexibility and individualised monitoring are essential to ensure/safeguard the holistic development of sports school student-athletes [92].

The student-athlete faces a unique set of challenges brought about by the simultaneous pursuit of both academic and sporting achievements [54]. Although most student-athletes at ‘Eden High’ found balancing sport and academics neither easy nor difficult (51%), 37% did describe it as difficult. This finding aligns to Ryba et al., [14], who demonstrated that balancing education and sport is a challenging proposition and one that is a concern for most high-performance student-athletes. The fact that some student-athletes at ‘Eden High’ stated that their academic workload interfered with their physical training correlates with previous research which demonstrates that the time commitments associated with participation in education alongside sports training can contribute to athletes prioritising education or sport with success in one venture coming at the expense of the other [93]. This data is supported with the RESTQ-52 data where conflicts/pressure was one of the highest stress scores. A potential solution to managing the varying schedules of sport-friendly school student-athletes and facilitating both education and sporting pursuits is program flexibility [54]. Coaches and teachers may identify and discuss period of heavy academic stress (examination timetables, coursework deadlines) or sporting commitments (e.g., major tournament or trials) so that student-athlete training schedules may be altered to provide sufficient time to revise/ complete assignments, or train for upcoming tournament or trials [54].

Student-athletes missed on average one academic lesson per week, whilst on some occasion’s student-athletes missed up to 12 lessons per week (although this is likely a special circumstance for an international athlete). This finding supports previous research, which highlights that missing school was an impact experienced by sports school student-athletes [29]. However, despite missing school and the challenges of combining high academic and sports workload, most student-athletes within ‘Eden High’ were achieving high academic grades (B, merit, or above). These findings are congruent with past research, which has indicated that athletes excel in both sport and education [94]. Such findings may correlate with the fact that most student-athletes at ‘Eden High’ displayed dual motivation towards both athlete and student roles. Furthermore, the findings may reflect the importance of the additional support offered by sports schools (e.g., extra tutoring, revision clinics, consistent check-ups from academic and sports staff) in protecting academic success [29]. Nevertheless, although 67% of student-athletes were somewhat to extremely satisfied with the academic support they received at ‘Eden High’, sport-friendly schools need to ensure that these academic support services are at optimal times for the student-athletes, are consistently offered across the school year and are encouraged, supported and advertised by the academic and sports staff. As such, whilst sport-friendly school student-athletes miss periods of school work, they must have appropriate resources (e.g., academic support programs) in place to overcome such negative impacts [29] and encourage student-athletes to maintain a dual motivation.

Alongside academic development, a sports-friendly school aims to develop athletic/physical performance. Overall, the majority of student-athletes within ‘Eden High’ felt they enhanced their physical, technical and overall sporting performance and rated their sports competence moderately to very competent. These findings are supported by previous research within DC and TIDS environments [95, 96], which suggest that through a multidimensional training programme, high volume/frequency of training, high-quality coaches and sports support staff, student-athletes develop their all-around athletic and physical performance. However, when comparing the sport competence and confidence scores with youth football academy players, ‘Eden High’ student-athlete scored lower (competence: 3.56 vs 3.92, confidence: 2.81 vs 3.39; [97]). Conversely, although physical competence was rated the lowest when comparing speed and power data from the physical fitness testing battery with two other sports schools in Qatar and Australia [98], ‘Eden High’ student-athletes were overall faster and more powerful, however this may have been due to the wide age range of the athletes in the current sample (e.g., 11.8–18.6 years; [98]).

Despite the positive impact ’Eden High’ may have on athletic and physical performance and competence, injury incidence was a potential negative impact of ‘Eden High’, which has equally been highlighted by previous sports school [29] and academy [99] research. The anatomical sites of injury may be a reflection of the sports represented (e.g., predominately invasion sports). The injury rate could be attributed to the high workload highlighted within the study. Some student-athletes at ’Eden High’ did not have a single rest day within their weekly schedule and although the RESTQ-52 data suggested that student-athlete were only stressed “sometimes”, the overall stress is higher than previous sports academy and school data (general stress: 2.23 vs 1.29 and 1.69, sport-specific: 2.21 vs. 1.56 and 1.64; [57]). Furthermore, when looking at the RESTQ-52 subscales, injury and fatigue were the highest stress scores and physical relaxation the lowest recovery score. Previous research has demonstrated that high workload is a key contributor in the accumulation of fatigue [93] and that student-athletes with higher weekly training loads have higher recovery-stress states than student-athletes with lower weekly loads [100]. However, within this study we cannot determine if the injuries are a function of participating in training and games regularly and at a high level and if injuries would be even higher without S&C and other support (e.g., physio). This should be addressed further in future research, where researchers explore the mechanisms behind these patterns and directly explore the correlation between high workload, rest, recovery and injury and how this differs between student-athletes.

Student-athlete within ‘Eden High’ developed other inter and intra personal skills (e.g., resilience, autonomy, social confidence, becoming role models). This development is supported by previous research, which suggests that sports schools encourage student-athletes to develop qualities and skills applicable in sports and other spheres of life [29]. Furthermore, student-athletes scored highest for communication, teamwork and social skills, which is unsurprising as student-athletes described an environment where they felt accepted, supported and included in peer groups. These findings are similar to previous literature where social skills were one of the main areas wherein sports school student-athletes showed individual development [101] and student-athletes tended to rate themselves significantly higher in the social domain than non-athletes [92]. Furthermore, ‘Eden High’ student-athletes’ overall LSSS scores are similar to that of British youth sports [102], sports high school and public high school [103] student-athletes (3.49 vs. 3.81, 3.81 and 3.85 respectively). As such, sport-friendly schools should continue to develop student-athletes technical, tactical, physical, academic and mental capabilities but additionally develop their personal, social and life skill capabilities [11], to ensure student-athletes develop transferable skills for life beyond the sport-friendly school environment [104]. Particularly because time-management and goal-setting were the lowest LSSS scores, sport-friendly schools may include workshops aimed at upskilling athletes in these specific areas [54].

‘Eden High’ may have provided many opportunities to retain and develop friendships within sport; however, time away from family and lack of friendships outside of sport and free time were typical consequences for student-athletes, a finding that aligns to previous sport school literature [29]. It appears within the qualitative data that there is a potential discrepancy between boarders and non-boarders and their feelings towards friends, family and free time. Boarders appeared to be more isolated from friends outside of school, had less freedom and found ‘Eden High’ more socially intense than non-boarders. Researchers have highlighted the risk of social isolation and feelings of alienation that result from spending substantial amounts of time away from family and inevitably having fewer opportunities to make and retain friendships outside sport [e.g., 105, 106]. As such, the time resources gained by eliminating travel time must not be used for additional training sessions/activities but should be reserved for the free, self-determined space/time necessary for personal and social development [107]. However, future research should directly explore the differences between boarders’ and non-boarders’ holistic impacts and the potential mechanisms that cause any discrepancy.

Finally, like previous studies [e.g., 108], this study demonstrated the potential influence physical activity and the combination of sport and academic workload has on student-athletes mental health. Double load (of school and sport) does not necessarily also lead to a double amount of (perceived) stress [109]. The physical load can be an effective recovery/relaxation for the mental side and vice versa, provided that the training for performance sports and academics are appropriately matched [109]. These findings align with previous research showing positive health benefits with increased physical activity and participation in sports [110, 111]. However, some student-athletes (e.g., those with injuries) found ‘Eden High’ challenging and stressful, taking a toll mentally and physically. As such, sport-friendly schools need to support student-athletes with mental health concerns through education, early identification of… and developing plans to recognise and refer student-athletes with psychological concerns [112]. Finally, body image is a concern at ‘Eden High’ with 17% of athletes identifying as at risk. This figure is similar to rates within adolescent athletes (16.3% [61]). Literature suggests that youth athletes, particularly girls, are becoming concerned about their body image at increasingly early ages [113] and body-related shame and guilt are increasing over time among female youth athletes [114]. However, this study did not distinguish between males and females. Future research should look at the discrepancy between gender across holistic impacts. Furthermore, these findings demonstrate that body image it a potential impact that should be carefully monitored within sport-friendly school settings [114].

## 5. Methodological reflections and future research

Whilst this study is the first study to assess the ‘in-time’ impacts and experiences of a sport-friendly school across all four areas of holistic athletic development using a mixed method research design, it is also important to be aware of its limitations. Some would argue that due to the first-hand experiences of the primary author, they already had their own preconceived ideas, potentially narrowing the analytic lens of the study. However, to mitigate against this, findings were related back to previous literature, critical friends (i.e., fellow research colleagues) were used and there were frequent peer-debriefing and critical reflections sessions between the co-authors, where the first author’s initial interpretations and proposed explanations were reviewed, discussed and challenged [115].

Due to the cross-sectional nature of this study (measurements collected at a single time point), where exposure and outcomes are simultaneous, it opposes the nature of ’transition’ as a process and the dynamic nature of sport-friendly school environments. Therefore, future research should address the calls for a longitudinal approach to investigate student-athletes development or changes over time, equally longitudinal data would be valuable in establishing causal explanations [116]. It is also important to track student-athlete from their initial transition into a sport-friendly school to identify against initial baselines how much the sport-friendly school actually influenced holistic development across time. Furthermore, the individual nature of student-athlete needs within a sport-friendly school will differ depending upon the sport, the academic pathway and the individual circumstances [91] and the data within this study are somewhat skewed by the student-athlete’s individual characteristics (e.g., existing life-skills, sports represented). Therefore, future research needs to consider the specificity of student-athlete antecedents and characteristics (e.g., sex, type of sport, age, development stage within the school, boarder vs non-boarder).

Another limitation is the use of self-report measures for assessing athletic/physical, academic, psychosocial and psychological impacts. While the participants’ perceptions were our main interest, there is a risk of social desirability affecting the answers [117]. Furthermore, due to the aim of this study, different questionnaires were needed in order to assess a wide range of holistic impacts. As a result, the length of the online questionnaire could have impacted the quality of the responses provided by the participants [118]. However, to mitigate against this the questionnaire was conducted in a quiet room, student-athletes were allowed sufficient breaks when required and were allowed to return to the questionnaire at a later time within the same day.

Given the complex and dynamic nature of DC environments [119], where student-athletes with varying characteristics have to interact with coaches, program culture and practices, then impacts are likely to vary across individuals and contexts [120]. As such, future research needs to seek further casual explanations (i.e., understanding of how the impacts have or have not been produced) to supplement the holistic athlete impact data (i.e., causal description). Furthermore, group averages do not allow for the investigation of inter-individual differences in holistic impacts of sport-friendly school student-athletes. As such, although this study used qualitative methodology to gain a more in-depth insight into the difference in program experiences and impact future research could employ different statistical techniques such as multilevel modelling or multivariate latent growth models (e.g. [121]). Finally, within this study, we evaluated one single sport-friendly school. As such, future research should explore and compare with other environments (e.g., academies, non-sport schools). This would then not only identify potential holistic impacts of sport-friendly schools but also address if and how these are distinctive from those of other settings or ‘normal’ adolescent development.

## 6. Conclusion

This study aimed to evaluate the ‘in-time’ impacts and experiences of UK sport-friendly school student-athletes across all four areas of holistic athletic development using a mixed method multidimensional research design. The findings showed that there are a multitude of positive impacts and experiences associated with being a sport-friendly school student-athlete. These positive impacts/experiences included: stress-relief/balance, high academic average, satisfaction with academic support, sport competence, all-round sport development, higher general and sport specific recovery than stress, life skill development, social support, positive peer and parent relationship, improved/stronger mental health, resilience and dual motivation. However, there are also areas of concern/high risk that sport-friendly school should be aware of. These include: addition of out of school sport, difficult balancing education and sport, academic lessons missed due to sport, injury, physical relaxation, fatigue, lack of free time, extra-curricular sacrifice, social intensity, sheltering from outside social experience, conflicts/pressure and body image. In summary, sport-friendly schools have the potential to promote many positive holistic impacts, however stakeholders need to be aware and mitigate the potential negative impacts observed in such programmes. Furthermore, there were large standard deviations and ranges across holistic impacts reflecting the highly inter-individual variation in student-athlete holistic impacts and experiences. As such, flexibility and individualised support and monitoring are essential features required of sport-friendly schools to ensure healthy and holistic development for all.
